# Smoking Gain?: Secondhand Smoke Exposure Influences Body Weight, Lipid Profiles in Offspring

**DOI:** 10.1289/ehp.117-a310a

**Published:** 2009-07

**Authors:** Victoria McGovern

**Affiliations:** **Victoria McGovern**, based in Durham, North Carolina, has written for *EHP* since 2000. She is a member of the National Association of Science Writers

An estimated 780,000 U.S. women continue smoking throughout pregnancy each year despite warnings about the elevated risk of birth defects in the children of female smokers. A new mouse study provides experimental evidence that exposure to secondhand cigarette smoke during pregnancy may lead to weight gain in offspring as well as changes in lipid profiles that may increase the chances of cardiovascular disease later in life **[*EHP*** **117:1042–1049; Ng et al.]**.

Oxidants in cigarette smoke have previously been shown to promote local and systemic inflammation and increase the risk of cardiovascular disease in both smokers and those exposed to secondhand smoke. Lipid oxidation in particular has been associated with cardiovascular diseases. Women have a higher risk of dying from cardiovascular disease than men and are more likely to die following a heart attack.

The current study may shed light on this observed sex-specific difference. Female pups of mice that were exposed to cigarette smoke for 4 hours a day, 5 days a week, throughout pregnancy grew up to have a higher body weight than their unexposed peers and had significant increases in plasma high-density lipoprotein (HDL), low-density lipoprotein (LDL), and total protein. However, these differences were not observed when the adult female offspring were fed a high-fat diet instead of a regular diet. On the other hand, smoke-exposed male pups gained more weight and displayed altered lipid profiles compared with their sex-matched, unexposed counterparts when they were fed a high-fat diet but showed little evidence of an effect of smoke exposure when fed a normal diet.

Although maternal exposure to cigarette smoke appeared to influence weight gain and lipid profiles in their offspring, lipid parameters in the dams themselves showed little change in response to smoke exposure. Additional work is necessary to understand why lipoprotein levels—which reflect cholesterol metabolism—are altered in the offspring in response to cigarette smoke exposure during pregnancy.

Abnormal body weight and dyslipidemia (abnormal plasma lipoprotein levels) are among the best-established risk factors for cardiovascular diseases. Although the new study does not investigate the mechanisms of the observed changes, it does suggest that prevention programs that emphasize avoidance of cigarette smoke during pregnancy could reap long-range health benefits for the newborn.

